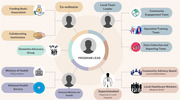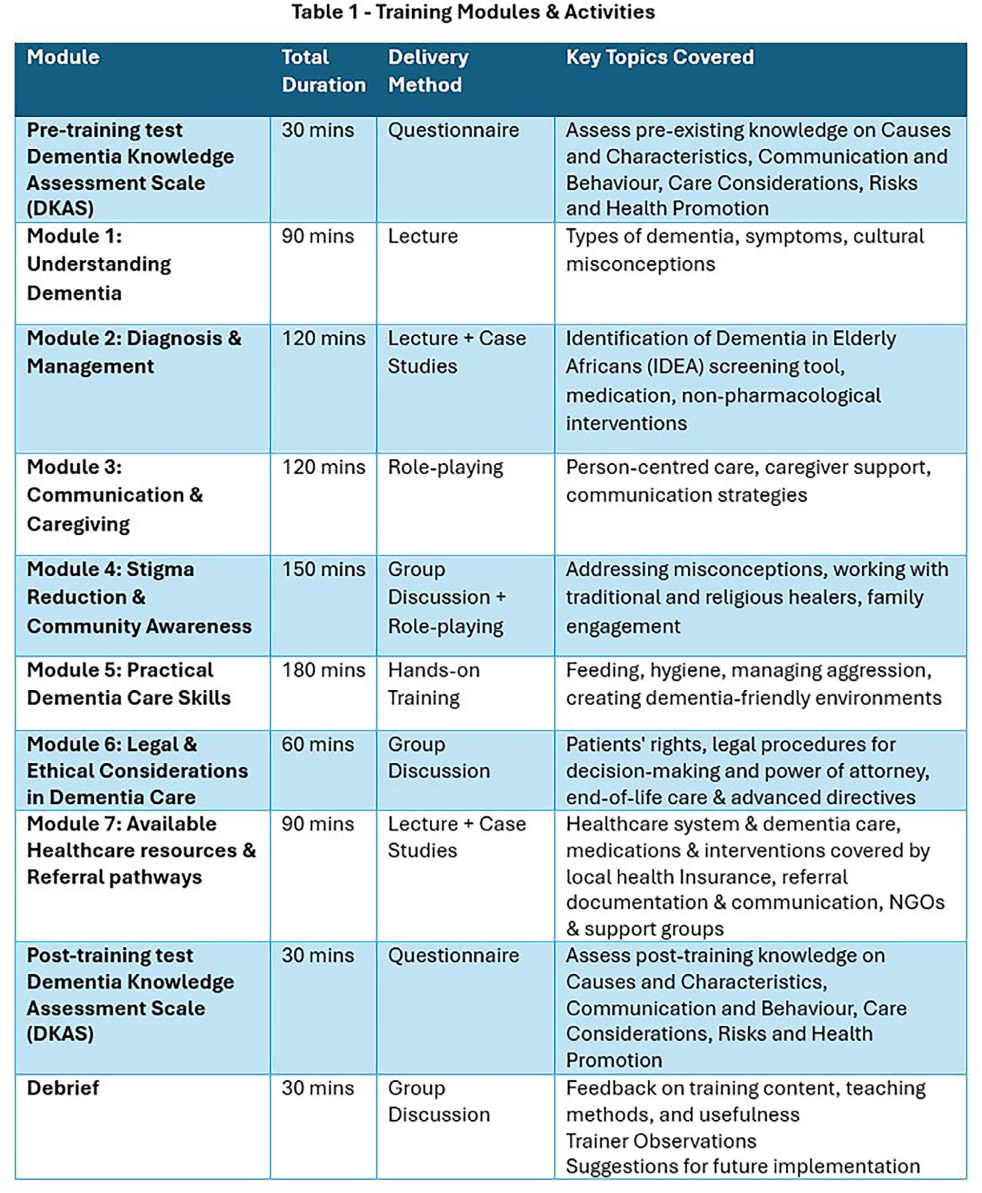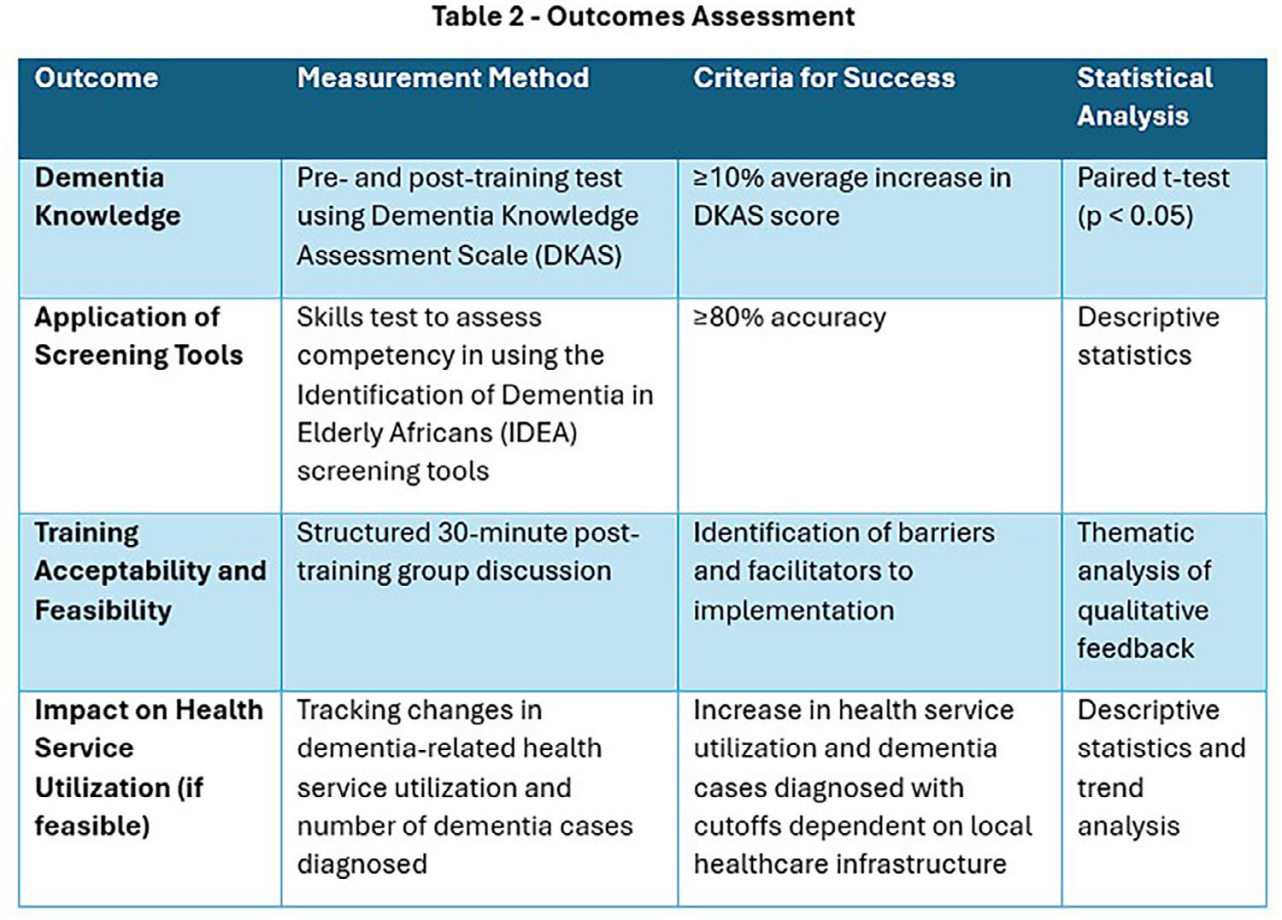# A Culturally Tailored Dementia Training Model for Primary Healthcare Workers in Sub‐Saharan Africa

**DOI:** 10.1002/alz70858_104740

**Published:** 2025-12-26

**Authors:** David Brodie‐Mends

**Affiliations:** ^1^ Korle‐Bu Teaching Hospital, Accra, Greater Accra, Ghana; University of California, San Francisco, SAN FRANCISCO, CA, USA

## Abstract

**Background:**

Dementia is a growing public health issue in Sub‐Saharan Africa (SSA), exacerbated by an aging population, limited healthcare infrastructure, and low awareness. In many SSA communities, dementia is often misunderstood or stigmatised, with affected individuals being viewed as witches or possessed. Additionally, many primary healthcare workers (PHWs) lack formal dementia training, impacting early detection, diagnosis, and care. This lack of knowledge contributes to the stigma, discouraging people from seeking help.

This protocol aims to address these gaps by providing a culturally relevant and evidence‐based dementia training model for PHWs. The goal is to enhance their competencies, reduce stigma, and improve dementia care. This model builds on successful frameworks, such as the WHO Mental Health Action Plan and the Dementia Awareness for Caregivers Initiative, adapting them to the unique healthcare and sociocultural context of SSA.

**Method:**

A structured governance and implementation framework (*Fig‐1*) ensures efficient execution, collaboration, and evaluation. The model is implemented in two phases:

*Phase 1: Community Advisory Board (CAB) Engagement and Needs Assessment*. A CAB consisting of PHWs (community health workers, nurses, general practitioners), caregivers, traditional and religious leaders is formed to conduct quantitative surveys: the Dementia Knowledge Assessment Scale (DKAS) assesses PHW baseline dementia knowledge, while the Dementia Attitudes Scale (DAS) evaluates local perceptions. Qualitative interviews and focus groups explore cultural beliefs, caregiving challenges, stigma, and training preferences. Stakeholder consultations: local dementia experts work with the CAB to adapt the curriculum to the local healthcare landscape, including referral pathways, insurance structures, and medication availability. Findings inform the development of a culturally tailored dementia training curriculum, integrating diagnostic, caregiving, and stigma‐reduction strategies.

*Phase 2: Training and Evaluation*. The curriculum is piloted using a train‐the‐trainer model. PHWs undergo a structured, multi‐day training program (individual sessions lasting 90 minutes or more, totalling over 8 hours) (*Table 1)*.

**Result:**

Outcomes are assessed by measuring changes in dementia knowledge and screening tool competency among PHWs as well as a group discussion (*Table 2)*.

**Conclusion:**

This mixed curriculum model ensures scientific rigor while incorporating local cultural insights for maximum impact. If successful, it can be scaled and adapted to other low‐resource settings.